# Artificial Intelligence for the Analysis of Biometric Data from Wearables in Education: A Systematic Review

**DOI:** 10.3390/s25227042

**Published:** 2025-11-18

**Authors:** Vittorio Meini, Lorenzo Bachi, Mohamed Amir Omezzine, Giorgia Procissi, Federico Pigni, Lucia Billeci

**Affiliations:** 1 Institute of Clinical Physiology, National Research Council of Italy (CNR), Via Moruzzi 1, 56124 Pisa, Italy; vittoriomeini@cnr.it (V.M.); lorenzobachi@cnr.it (L.B.); giorgiaprocissi@cnr.it (G.P.); 2 Information Systems, Grenoble École de Management, 38000 Grenoble, France; mohamed.omezzine@grenoble-em.com (M.A.O.); federico.pigni@grenoble-em.com (F.P.)

**Keywords:** wearables, artificial intelligence, education, personalized learning, biometrics

## Abstract

Wearable devices provide reliable biometric measurements in different contexts, and AI algorithms are increasingly being used to analyze this data. The objective of this review is to examine the use of wearable devices to collect biometric data combined with AI algorithms in an educational setting. A systematic review was conducted through the PRISMA methodology, by searching the Scopus database for works that included wearables, biometrics, and AI algorithms. A total of 43 studies were included and examined. The objectives, the type of collected data, and the methodologies of the included studies were investigated. Most articles utilized machine learning and deep learning algorithms for classification tasks, such as detecting stress or attention. Other applications included human activity recognition (HAR) for classroom orchestration and emotional or cognitive state detection. Many of the studies applied knowledge from previous works to the educational context, resembling exploratory research. Conversely, some authors developed tasks and methodologies tailored to the educational context. The strengths and weaknesses of the presented studies were discussed to propose future research directions. The main findings of this review highlight the advantages of the combination of multimodal sensing and predictive modeling in education with the eventual prospect of personalization. The absence of standardized acquisition and reporting remains the main barrier to replication, benchmarking, and synthesis across studies.

## 1. Introduction

Wearable devices allow biometric measurements in a cost-effective and user-friendly manner, with sufficient reliability [1]. For these reasons, these devices are now applied in various contexts, including sports medicine [2], cardiovascular care [3], and autism behavioral therapy [4], among others.

Commercial wristbands (like Google Fitbit, ScanWatch, Empatica E4) can provide electrocardiography (ECG), allowing the computation of heart rate (HR) and heart rate variability (HRV), galvanic skin response (GSR), as well as peripheral oxygen saturation (SpO_2_), skin temperature, inertial and motion data measured by accelerometers and gyroscopes. Typically, wristbands combine multiple light sources to measure the photoplethysmogram (PPG) signal, providing an estimation of blood volume pulse (BVP). Furthermore, some devices, available to the public, such as BrainLink (Macrotellect, China), Muse (InteraXon, Canada), and EPOC (Emotiv, USA), can measure EEG signals.

Some of these signals (i.e., EEG, HR, HRV, GSR, and ECG) are widely used in the detection of anxiety and psychological stress [5,6]. Wearables play an important role also in the educational context, where academic stress is widespread, especially after the COVID-19 pandemic [7]. Hernández-Mustieles et al. in their review [8] highlighted several applications of wearables in education, such as stress detection, improvement of learning efficiency, development of a students’ authentication system, and helping students with intellectual disabilities. Using cameras, accelerometers, and gyroscopes integrated in wearable devices, the authors of [9] developed an algorithm for students’ attention detection. In [10], accelerometer sensors were employed, along with audiovisual data and eye-tracking, for the extraction of the orchestration graphs of several classrooms. The authors of [11] utilized motion sensors, such as accelerometers and gyroscopes, for human activity recognition (HAR).

Non-wearable, computer vision-based pipelines can enable automated assessment of a subject’s action from commodity videos and shared repositories [12,13]. Vision-based models are characterized by lower entry costs, higher scalability, and support reproducibility for classroom and simulation analytics using open data. Yet, the use of wearables is desired when the construct involves an evaluation of the broader physiological state and demands continuous monitoring. Since it is known that personalization of education is beneficial to academic performance and the wellbeing of students [14], usage of biometric data represents an additional and potentially very effective tool in improving the education delivery for all subjects involved. Moreover, due to their nature and to the provided data, wearables can be useful in monitoring students’ performance during physical education classes, e.g., in [15]. Authors proposed an educative “exergame” called “Running Otello 2” to third-year students of primary education schools, while monitoring movements and heart rate of the participants. Heart rate data was collected to estimate the efficiency of exercising, and the authors concluded that, as a future development, HR could be used to customize the difficulty level of the exercise.

The authors of [16] used a VU-AMS device to collect EEG, ECG, GSR, blood pressure (BP), and impedance cardiogram (ICG) to classify emotions of students during a bug-hunting game. Then, the authors trained two deep-learning algorithms: the first one is a simple binary algorithm that returned an accuracy of 84.4%, and the latter is a four-emotions model which obtained a lower accuracy of approximately 57%.

Recently, wearables have been combined with artificial intelligence (AI) algorithms [5,9,10,11,16], such as machine learning and deep learning methods (linear regression; random forest; support vector machines; naïve Bayes; convolutional neural networks—CNNs; long short-term memory—LSTM), mainly to perform classification tasks.

Shoaib et al. [11] selected three classifiers (naïve Bayes, decision tree, and kNN) to recognize complex human activities. The proposed experimental setup included the following activities: walking, jogging, biking, walking upstairs, walking downstairs, sitting, and standing. In [9], the J48 decision tree learning algorithm was used to create a framework for detecting students’ attention, based on visual inputs of their activities and motion sensors (accelerometer and gyroscope). Prieto et al. [10] used eye-tracking data, accelerometer data, audio recordings, and video recordings to extract a classroom orchestration graph through an LSTM network. Silvis-Cividjian et al. [16] also used LSTM for their emotion recognition task.

This study has been performed in the context of the “DIGital skills for transformative Innovation Management and Entrepreneurship (DIGI-ME)” [17] project, which introduces a novel framework for personalizing students’ learning paths. This approach involves assessing students’ digital, psychophysiological, and physiological profiles over time. This multidimensional data collection uses validated instruments and wearable sensors to provide insights into customized learning pathways. AI tools will be used to analyze multimodal data and promote personalized learning. A web-accessible user interface will be implemented to enable continuous monitoring and adaptation via a configurable dashboard. This platform will enable career mentors and researchers to visualize and track comprehensive student profiles throughout their academic courses.

It should be noted that AI models, together with different types of signals such as the ECG, have been used for biometric identification [18]. Collecting data that could be potentially linked to a specific person calls for rigorous handling practices, even in applications that do not have the primary goal of biometric identification, such as AI-assisted education, while a description of these privacy-preserving practices is out of the scope of this review, more can be read here [19]. Additionally, while non-ethical uses of AI models have been reported, the applications of AI-assisted education are most often intended to be used as a tool of the teacher/professor. As such, through their professional experience, they can overlook any output they deem unreasonable or harmful. Nonetheless, building AI models should be performed in an ethically aware fashion, using the known practices and methods to minimize risks [20].

A recurring issue found in existing literature is the lack of a structured framework for applying wearables in an educational context. An approach that combines wearables with AI algorithms could offer interesting strategies for developing personalized learning pathways. To the best of our knowledge, this review is differentiated from similar work in the analysis of the intersection of AI algorithms, wearable biometric signals, and educational context.

This review aims to address this problem, investigating the state of the art about the use of AI algorithms to analyze biometric data collected through wearables in an educational context, underlying applications, and limitations of this procedure. In particular, eventual applications to personalized learning will be debated.

The rest of this article is divided as follows: Section 2 explains the methodology used in this review; Section 3 offers an overview of articles included in the analysis, their objectives, sample size, collected data, used devices, task proposed, AI tools applied and best metrics for each article; Section 4 discusses the results of this review with a focus of applications of wearables combined with AI for personalized learning and the role of datasets. Appendix A incudes a brief glossary of selected psychological and cognitive terms used throughout the manuscript.

## 2. Materials and Methods

This systematic review was performed using PRISMA (Preferred Reporting Items for Systematic Reviews and Meta-Analyses) methodology [21].

This systematic literature review originates from the following research questions:

RQ1 *How can biometric data collected via wearable devices and analyzed through AI algorithms provide reliable information in educational contexts?*
RQ2 *How can these frameworks enable continuous personalization in education?*

State-of-the-art studies were investigated for contributions that included:

1. Wearable sensors that are are unobtrusive and accessible;
2. Biometrics;
3. AI algorithms;
4. Stated in the abstract or introduction that the scope of the paper was within education.

The prospective efficiency of the proposed computational methods will also be considered.

The research papers were gathered in May 2025 within the Scopus database through the following query:

(TITLE-ABS-KEY (“AI” OR “Artificial Intelligence” OR “Machine Learning” OR “Deep Learning” OR “Reinforcement Learning” OR “Neural Network*”) AND TITLE-ABS-KEY (“wearable” OR “wearable device*” OR “wearable sensor*” OR “wearable technology*” OR “smart wearable*” OR “biometric wearable*”) AND TITLE-ABS-KEY (“education*” OR “school*” OR “college*” OR “universit*” OR “lecture*” OR “student*” OR “learning environment*” OR “classroom*” OR “teacher*” OR “curriculum*” OR “pedagog*” OR “Intelligent tutoring system”) AND NOT TITLE-ABS-KEY ((“health*” AND NOT “mental”) OR “medicine*” OR “medical*” OR “patient*” OR “clinical*” OR “rehabilitation*” OR “therapy*” OR “sport*” OR “fitness*” OR “nursing*” OR “physiotherapy*”))

**Figure 1:**
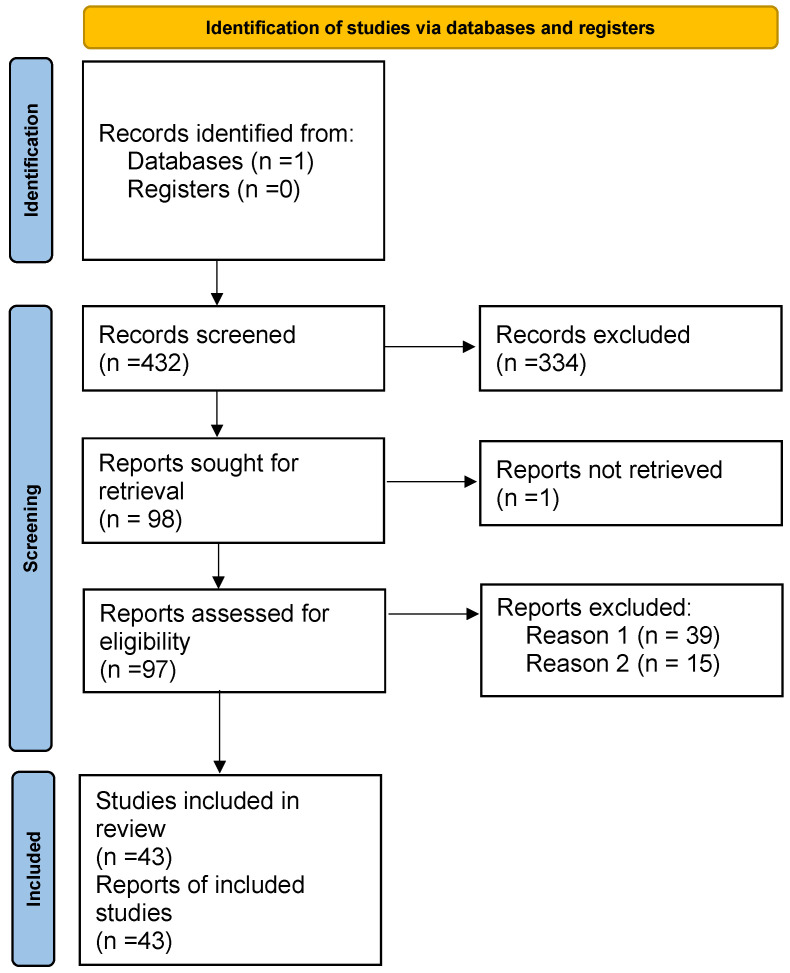
PRISMA flowchart. After the screening, 43 studies were selected to be included in the review.

The PRISMA methodology includes three steps: identification, screening, and reviewing the included studies. As shown in Figure 1, 432 records were identified by searching the Scopus database. During the initial screening, 334 records were excluded based on the title and abstract. The remaining 98 reports were sought for retrieval; however, one of them could not be retrieved. The 97 reports were then assessed for eligibility, and studies that met the following criteria were excluded:

a. The authors did not collect biometric data (n=39).
b. The study did not involve AI algorithms (n=15).

A total of 43 studies were included in the review.

Two authors consulted the Scopus database and wrote the above query. Discussion among the authors was crucial in deciding whether to include specific studies. Accuracy and the F1 score are considered the most appropriate metrics for evaluating the quality of the studies because these metrics are extensively used in the literature. Additionally, the ROBINS-I tool for risk of bias assessment [22] was adapted to the scope of this review and applied to the included studies. Four ROBINS-I domains were considered: intervention classification, missing data, outcome measurement, and results selection. Bias in interventions can be due to self-reported data or tasks that are arbitrarily labeled. Bias due to missing data occurs when a device’s signal contains noise that cannot be addressed through preprocessing. Bias in outcome measurements is assessed by analyzing the clarity of the pre-processing of data described in the study. Selection bias occurs when only the results related to the best-performing algorithm are reported.

## 3. Results

### 3.1. Characteristics of Included Studies

Table 1 shows the following characteristics for each included article:

- Objective of the study;
- Sample size and description;
- Collected data and used devices;
- Proposed task;
- Tools and models used, including AI algorithms and datasets;
- Best accuracy score;
- Best F1 score.

**Figure 2:**
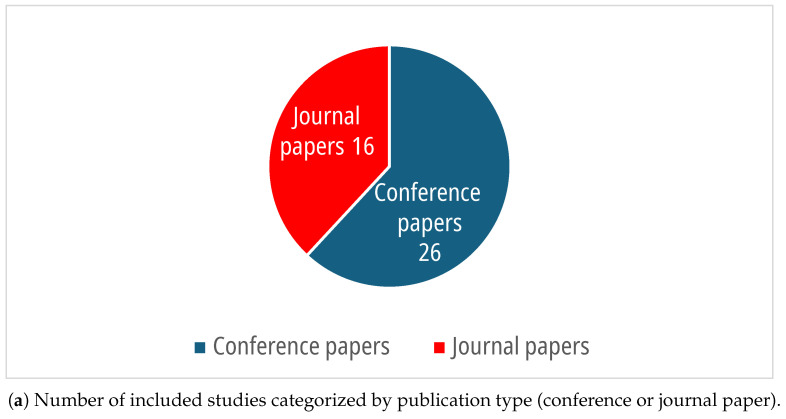

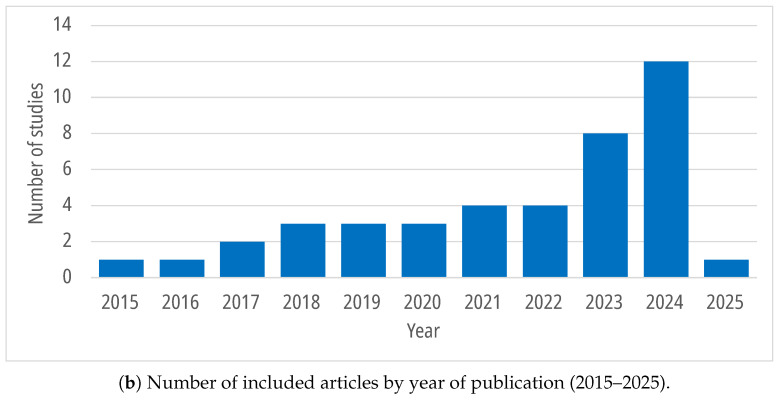
Overview of included studies by publication type and year.

As can be noticed in Figure 2, the papers included in this review are, for the most part, conference proceedings and were published in the last few years. No constraints on publication date were imposed in the searching phase. The oldest article dates back to 2015, and the number of studies related to the considered topics has increased in the last few years, especially since 2022. The higher prevalence of conference papers could be an indication of the novelty of this field, which appears to be at an early stage.

### 3.2. Objectives and Tasks

**Figure 3:**
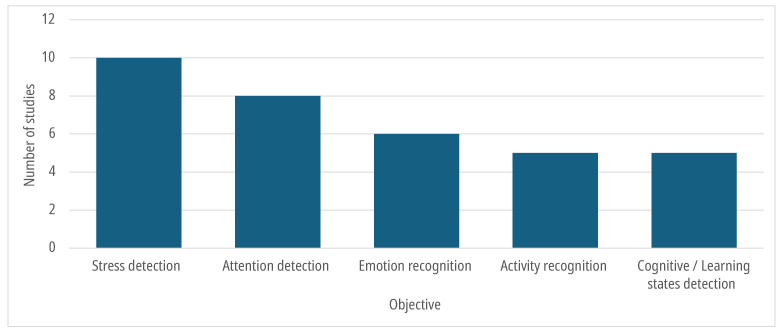
Number of included articles by objective, considering that each article may have several objectives.

In the considered articles, AI algorithms were mainly used for classification or detection purposes, as shown in Figure 3. Stress and attention levels are the most assessed factors in the articles, and biometric data are widely used for their detection. To label stress levels, multiple methods can be used; for instance, in [23], the authors administered self-assessment surveys to participants to label the data.

In [24], students were asked to solve a sudoku exercise in three different scenarios, filling a questionnaire at the end of each trial where they self-assessed their stress level. In the first scenario, the student was left alone in the room, being exposed to horror or discordant audio and watching a horror video; in the second one, a person observed the participant during the task; in the last one, the participant was exposed to comforting audio and videos. The sample was split into two groups of 15 students: the first group solved a medium-difficulty Sudoku puzzle, while the second group faced a hard-difficulty puzzle. The authors aimed to investigate how difficulty level and scenario impact stress levels, and to detect each participant’s stress level from their biometric data.

**Table 1:** Characteristics of included studies.

Study	Objective	Sample	Data and Devices	Task	Tools	Best Accuracy	Best F1
[25]	Concentration estimation	13 students at a Japanese university	Accelerometer and gyroscope data (MetamotionS), heart rate (Fitbit), face orientation and eye gazing (Webcam)	Two video lectures watching on Youtube. Participants report their feelings every 90 s	Gradient boosting, decision tree, logistic regression, random forest, and SVM (classification)	74.4% Random forest with user-dependent-cross-validation	Not provided
[26]	Affective and motivational states measurement	22 graduate and undergraduate students in Australia	EEG along with performance metrics (Emotiv EPOCX), eye tracking (Tobii Nano Pro), GSR (Empatica E4)	Pretest on the previous knowledge, essay reading and writing, post-task assessment	ConvTran (classification)	Metacognitive processes: 74.1% (EEG performance metrics), low cognitive processes: 91.5% (EEG), high cognitive processes: 92.2% (EEG)	Metacognitive processes: 73.5% (EEG performance metrics), low cognitive processes: 91.5% (EEG), high cognitive processes: 92.2% (EEG)
[24]	Stress level detection	30 students at several universities	PPG (Polar variety sense), ECG (BMD101), EEG (Mindwave Mobile)	Sudoku solving task, divided in three scenarios, followed by self-assessment of stress level	StressNeXt, LRCN, self-supervised CNN (classification)	93.42% LRCN with ECG data	88.11% LRCN with ECG data
[27]	Activity recognition	8 neurodiverse students	Accelerometer and gyroscope data, heart rate (Google Wear OS)	Reading and follow up Q&A, typing, prompt writing, reading and follow up Q&A	Logistic regression, MLP, CRNN, single LSTM, federated multi-task hierarchical attention model (FATHOM) (classification)	97.5% CRNN, Leave-one-out cross-validation	91.8% FATHOM, Leave-one-out cross validation
[28]	Cognitive states detection (focused attention and working memory skills level)	86 undergraduate students	EEG (Emotiv EPOC)	Cognifit test, that stimulate perception, memory, attention, and other cognitive states	Logistic regression (feature selection), NN, linear SVC (classification)	90% linear SVC, focused attention	Not provided
[29]	English communication enhancement	Not provided	Temperature sensors, blood pressure sensors, pulse oximeter, heartbeat sensors, ECG sensors, EEG sensors	Not provided	kNN, NB, SVM, SVM with an improved satin Bower bird optimization algorithm (SVM-ISBBO) (classification)	92.34% SVM-ISBBO	Not provided
[30]	Attention and interest level detection	30 students	PPG, acceleration, and gyroscope data (second generation Moto 360 smartwatch)	Two lectures, followed by administration of a questionnaire	Decision tree, NN, SVM, naïve Bayes (classification)	98.99% Decision tree, interest level, 95.79% SVM, difficulty level	Not provided
[31]	Activity recognition (reading/relaxing with open eyes)	14 college students	EEG (Muse portable brainwave reader)	MATH, SHUT (eyes), READ (and answer test), OPEN (relaxation with open eyes)	K-means (classification)	71% K-means (K = 12)	Not provided
[32]	Teacher activity and social plane prediction of interaction	One teacher	Eye tracking, EEG, accelerometers, subjective video and audio	Lecture simulation: explanation, questioning, group work, whole-class game	Random forest, SVM, gradient boosted tree (classification)	67.3% teacher activity, random forest (Markov chain, top 80 features), 89.9% social plane, gradient boosted tree (top 81 features)	Not provided
[33]	Classification of learning events, personalized learning system implementation	15 healthy participants	EEG (Emotiv EPOC), Oculus	Wisconsin Card Sorting Test (WCST) (classification), 2D video watching, 3D video watching, questionnaire administration (personalization)	SVM (Gaussian kernel), CNN, deep spatiotemporal convolutional bidirectional LSTM network (DSTCLN) (classification), Q-learning (personalization)	84.81% DSTCLN	Not provided
[34]	Learning states and learning analytics analysis	Two groups: 32 third-year high school students and 20 first-year high school students in Hong Kong	Heart Rate, calories consumption, accelerometer and gyroscope data (Fitbit Versa)	Wearing a smartwatch during school time and, preferably, all the time for one week. Reporting learning activities periodically through a mobile app	LSTM, hybrid algorithm integrating LSTM and CNN (classification)	95.6% LSTM	80% LSTM
[35]	Computing heart rate variability from heart rate and step count	25 university students, Auckland	HRV ECG-based (Polar H10), HR PPG-based (Fitbit Sense)	Three days monitoring on weekdays from 9 am to 4 pm. Answering a questionnaire about worry, stress, and anxiety	Naïve Bayes, linear and logistic regression, decision tree, random forest, LSTM (classification)	Not provided	Not provided
[36]	Attention level prediction	18 students aged 12–15 of a middle school in Chongqing, China	BVP, IBI, GSR, skin temperature (Empatica E4), EEG	Learning video watching, student action recording	SVM, decision tree, random forest, naïve Bayes, Bayesian network, logistic regression, kNN (classification)	75.86% SVM	70.1% SVM
[37]	Attention level detection	100 participants	EEG (Neurosky device)	Video lesson	CART, XGBOOST (feature selection), K-means (clustering), SVM linear kernel, logistic regression, ridge Regression (classification)	91.68% SVM	91.53% SVM
[38]	Learning immersion experience evaluation	37 college students in China	VR glasses (Pico Neo 2), EEG (BrainLink headband), PPG (KS-CM01 finger-clip)	Questions reading without answers, VR video about the city of Guilin and online teaching video on English words, questionnaire administration	SVM-RBF (radial basis function) (classification)	89.72% SVM	Not provided
[39]	Self-assessed concentration detection	16 students from Haaga-Helia University of Applied Sciences in Helsinki	HR, GSR, skin temperature, accelerometer data (Empatica E3)	Wearing device during home study, self-reporting concentration through mobile app	Boosted regression tree, CNN (classification)	99.9% Boosted regression tree, pseudo-labeled set	Not provided
[40]	Fatigue level detection	23 healthy undergraduate students	BVP, GSR, EEG-related features (Empatica E4)	Test Auditory Odball (AO)	Random forest (feature selection), multiple linear regression (classification)	91% MLR	Not provided
[23]	Stress detection for autistic college students	20 (10 neurotypical, 10 autistic) college students in the USA	Heart Rate, sleep, GSR, temperature and accelerometer (Fitbit), step count, GPS location, sound intensity and light data (phone sensors)	Pre-interview, wearing Fitbit during regular lives activities for at least one week, post-interview	Information Sieve algorithm (to label unlabeled data), logistic regression, kNN, SVM linear kernel, NN (classification)	70% SVM	Not provided
[41]	Perceived satisfaction, usefulness, and performance estimation	31 university students forming 6 groups	GSR, BVP, HR, skin temperature (Empatica E4)	Wearing device during each class, survey filling	GSR explorer (noise removal), random forest, SVM with linear, radial and polynomial kernels (classification)	Not provided	Not provided
[42]	Emotional state detection	30 people from lectures and/or workshops in China	Heart rate, acceleration	Wearing device during 5 days of lectures/workshops	Decision tree, kNN, logistic regression, random forest, multilayer perceptron, SVM with linear, radial and polynomial kernels, gradient boost, XGBoost, LSTM (classification)	Activation: 89.53% random forest. Tiredness: 95.14% gradient boosting. Pleasant feelings: 91.65% random forest, gradient boosting. Quality: 93.13% gradient boosting. Understanding: 93.80% XGBoost	Not provided
[43]	Stress detection	9 participants	GSR (custom-built device), heart rate (LG smartwatch and Polar H7)	Hand in ice (S), singing (S), game (S), stroop (S), math (S), light conversation (NS), homework (NS), emails (NS), eating (NS)	Correlation-based feature subset evaluation (feature selection), naïve Bayes, SVM, logistic regression, random forest (classification)	Not provided	Intended stress: 59.2% naïve Bayes. Self-reported stress: 78.8% random forest
[44]	Emotion detection	4 students	Heart beat, step count (Xiaomi MIband 1 S)	Wearing Xiaomi MIband for different time	SVM (classification)	Fusion model: 92.02% user 1, 94.07% user 2, 93.36% user 3, 96.81% user 4	Not provided
[45]	Degree of retention and subjective difficulty detection	8 healthy males among college students and social workers	Eye potentials, acceleration, and angular acceleration (JINS MEME), body temperature, RRI, LF/HF, HR, accelerations (MyBeat)	From TOIEC: 210 English vocabulary questions, self-reporting degree of retention and subjective difficulty	Not provided	81% LOSO and Cross-validation	Not provided
[46]	Activities monitoring	44% of a total of 18 undergraduate students of Computer Engineering	Accelerometer and gyroscope data, heart rate, pedometer, skin temperature, and calories (MSBand)	Activities monitoring for 8 weeks, self-report by the participants	MLP, naïve Bayes, J48, random forest, JRIP (classification)	87.2% random forest	Not provided
[47]	Stress level recognition	10 students of the Faculty of electrical engineering Tuzla	ECG, GSR	Relax, oral presentation, written exam	SVM linear kernel, linear discriminant analysis, ensemble, kNN, J4.8 (classification)	91% SVM ECG and GSR	Not provided
[48]	Perceived difficulty level recognition and success prediction	27 individuals	EEG (Emotiv EPOC), ECG, EMG (Shimmer v2)	English Text, 20 questions from Oxford Quick Placement Test	kNN (K = 1, 3, 5) SVM, linear and radial basis function kernel (LSVM, SVM-RBF), linear discriminant analysis (LDA), decision trees (DT) (classification)	81.92% LSVM EEG-MFCC [0.5–40] mel frequency cepstral coefficients	74.21% LSVM EEG-MFCC [0.5–40]
[49]	Critical thinking detection	Engineering undergraduate students	EEG (Muse headband)	Detecting false and irrelevant information from a video	SVM (linear, quadratic, cubic, medium Gaussian, coarse Gaussian), kNN, NB, decision tree (classification)	100%	Not provided
[50]	Stress classification	23 engineering students	EEG (Emotiv EPOC), GSR, skin temperature, HR (Empatica E4)	MIST (Montreal Imaging Stress Task)	Random forest, kNN (classification)	99.98% random forest	Not provided
[51]	Stress detection	21 participants of an algorithmic programming contest	Acceleration, PPG, GSR, skin temperature (Empatica E4)	Wearing device during free day, lectures and contest session	PCA anda LDA, PCA and SVM (radial), logistic regression, random forest, multilayer perception (classification)	92.15% logistic regression (HR and GSR), multilayer perception (HR, GSR, and ACC)	Not provided
[52]	User/device recognition, class/break recognition, estimating self-reported affect and mood state	42 students and 2 professors from University of Italian-speaking Switzerland	GSR, BVP, acceleration, skin temperature (Empatica E4), heart rate derived from BVP	Wearing device during 26 classes (including exams), self-reporting lifestyle habits	Random forest, light gradient boosting machine (LGBM), spectro-temporal residual network (STResNet) (classification)	56.63% STResNet user/device 90.8% LGBM class/break	49% STResNet user/device 72% STResNet class/break
[53]	Cognitive state detection	127 undergraduate university students each day for 6 weeks)	EEG (Emotiv EPOC)	Cognifit test	Logistic regression (LR), NNs, SVMs, random forest, LSTM, ConvLSTM (classification)	RF: engagement (92.1%). LR: instantaneous Attention (95%), focused Attention (98%), working Memory (94%), visual Perception (95%), NN: planning (95.6%), shifting (95.6%)	Not provided
[54]	Physical, social and cognitive stressor identification	26 university students	ECG (smartshirt), HRV extracted from ECG (Kubios Scientific software, unknown version), timestamps of activities (Empatica E4)	Cold pressor (physical), Trier Social Stress Test (social), Seated Stroop task (cognitive), State-Trait Anxiety Inventory (self-reported state anxiety)	SVM with linear kernel, random forest trees, naïve Bayes, kNN (classification)	79.1% SVM (multi-class, 10-fold CV)	Not provided
[55]	Student grades prediction, considering the students’ stress factors	10 students, augmented to 7680 students through data augmentation	GSR, Skin Temperature, Heart Rate (Empatica E4)	Students wore the device during three exams	Physionet dataset, CNN, decision tree regressor, support vector regressor (SVR), KNN regressor, random forest regressor (classification)	Not provided	Not provided
[56]	Developing an LSTM-based emotion recognition system	30 participants	Respiration, GSR, ECG, EMG, skin temperature, and BVP	Watching relaxing, boring, amusing, and scary videos	CASE dataset, LSTM (classification)	Not provided	95.1% LSTM, incorporating all eight sensing modalities
[57]	Stress level analysis	10 university student	Physionet dataset, GSR, Skin Temperature (Empatica E4)	Students wore the device during three exams	SVM, KNN, 10-fold cross-validation (classification)	70% KNN	80% KNN
[58]	Providing educators with real-time insights into student engagement and cognitive responses	Not provided	Eye-tracking, typing behavior, heart rate, GSR, mouse movements, and click pattern	The data were collected during online exams	Distributed machine learning (DML), Residual network (ReSNet) (classification)	85.7% ResNet + DML	Not provided
[59]	Prediction of depression, stress, and anxiety	700 students at Notre Dame university in 2015–2017 period, dropped to 300 in the 2017–2019 period	Step counts, active minutes, heart rate, sleep metrics (Fitbit), bad habits, personal inventory, education, exercise, health, origin, personal information, sex, and sleep (self-reported survey)	Data collection during academic life	NetHealth dataset, Multitask learning (MTL), random forest, XGBoost, LSTM (classification)	Not provided	Not provided
[60]	Emotion recognition	15 participants aged between 24 and 29	GSR, respiration, skin temperature, weight	Exposition to four distinct emotional states: baseline, stress, amusement, and meditation, all of which were labeled accordingly	WESAD dataset, recursive feature elimination in random forest (REF-RF), through 10-fold cross-validation (feature selection), EmoMA-Net (classification)	99.66% EmoMA-Net	98.43% EmoMA-Net
[61]	PPG data generation	10 university student	PPG signal	Students wore the device during three exams	Physionet dataset, conditional probabilistic auto-regressive (CPAR) model (classification)	Not applicable	Not applicable
[62]	Stress detection	15 participants aged between 24 and 29	ECG, GSR, EMG, respiration, skin temperature, and three-axis acceleration (RespiBAN), blood volume pulse (BVP), GSR, body temperature, and three-axis acceleration (Empatica E4)	Exposition to four distinct emotional states: baseline, stress, amusement, and meditation, all of which were labeled accordingly	WESAD dataset, extra tree classifier (feature selection), XGBoost, fine-tuning (classification)	Not provided	96% fine-tuned XGBoost
[63]	Engagement recognition across classrooms, presentations and workplaces, under a unified methodological framework	24 university students (SEED dataset), 10 audience member across multiple presentations (APSYNC dataset), 14 academic workers (Workplace dataset)	GSR (Empatica E4)	Wearing the device during nine lectures (SEED database), presentations (APSYNC dataset), and various tasks over 28 days (Workplace dataset)	SEED dataset, APSYNC dataset, Workplace dataset, 27 of machine learning models, 5-fold cross-validation, Leave Out Participant Out (LOPO) and Leave One Session Out (LOSO), single-dataset training, multi-dataset training, Leave-One-Dataset-Out (LODO) cross-validation, impurity-based feature importance analysis (classification)	93.4% APSYNC dataset, single-dataset training, 5-fold validation	Not provided
[64,65]	Emotion recognition into human–computer interaction	15 participants aged between 24 and 29 (WESAD), Not provided (self-collected dataset)	ECG	Exposition to four distinct emotional states: baseline, stress, amusement, and meditation, all of which were labeled accordingly (WESAD), Not provided (self-collected dataset)	WESAD dataset, CNN (classification)	87.90% (WESAD)	87.71% (WESAD)

However, the most common practice is to measure biometric signals while students are engaged in activities with a fixed level of stress. For instance, in [43] activities were classified in a binary manner (stress vs. non-stress), whereas authors of [51] provided three different stress scenarios during an algorithm programming contest assigning a value to each scenario; free days, related to the lowest stress level, were labeled as 0, lectures were labeled as 1 and contest sessions were labeled as 2. Similarly, the authors of [47] used algorithms to detect three different stress contexts: relaxed state, oral presentation, and exam.

Validated tests can also be used as a baseline for the level of stress assessment. The authors of [50] used the Montreal Imaging Stress Test (MIST), consisting of an initial rest phase, followed by the submission of some arithmetical task (control phase), and the last phase featuring a similar task with the addition of a stressor element. In the experimental phase, the average score of the other participants was shown on the monitor during the task. In [54], the Trier Social Stress Test (TSST) was used to investigate social stress. The first phase consisted of the preparation and exposition of a short speech; the second phase consisted of arithmetic tasks, followed by a final relaxation phase.

Regarding attention recognition, in [37], data were labeled assuming that the α and β bands of EEG are related to attentive states and the δ, θ, and γ bands are related to inattentive states. The level of attention can also be evaluated by using a self-report tool [25,30,39], such as questionnaires, interviews, or a web app, or recording students’ actions [36].

For example, the subject’s attention level can be investigated through the Cognifit test [28,53], which is used to stimulate perception, memory, and other cognitive states.

Several AI algorithms have been used to classify the level of attention. For example, in [26], the authors used a convolutional model called *ConvTran*, a deep learning algorithm proposed in [66] to classify time series data, predicting metacognition (orientation, planning, evaluation, monitoring), low cognition (first-reading, re-reading), and high cognition (elaboration, organization) processes.

In human activity recognition, biometric data are often collected along with data from accelerometer and gyroscope sensors provided by smartwatches. For instance, in [46], acceleration and gyroscope data were used together with biometrics to recognize different activities: eating, running, sleeping, classroom session, exam, job, homework, transportation, watching TV series, and reading.

The study described in [52] applied an AI algorithm and acceleration data for class vs. break recognition. Moreover, the authors aimed to detect the emotional states of the participants in terms of positive activation, negative activation, and valence, which were labeled through self-reporting. In [42], participants were asked to answer three questions about their activation, tiredness, and pleasant feelings: researchers asked participants to assess these parameters on a Likert Scale between 0 and 2 and trained several AI algorithms to detect each of these emotions, including the decision tree, kNN, logistic regression, random forest, multilayer perceptron, SVM, gradient boost, XGBoost, and LSTM. In [44], instead, researchers asked participants to manually label their emotions (positive, negative, and neutral) whenever their emotions changed and trained an SVM for emotion recognition.

In [55], the authors aimed to predict students’ grades through machine learning while considering their stress factors. The authors performed data augmentation on the Physionet dataset, expanding the sample size from 10 students to 7680 students. Students were labeled as “stressed” or “not stressed”, according to arbitrary biometric thresholds applied by the authors. Then, if a student was labeled “stressed,” their grade was predicted to be between 0 and 80; otherwise, it was predicted to be between 80 and 100, while the study presents an interesting approach, it lacks reliability and is susceptible to bias, as discussed further in Section 3.3.

The main goal of the study in [35] was to compute heart rate variability (HRV) and predict higher-stress time intervals from heart rate and step count. The authors periodically collected feedback from students about their stress, worry, and anxiety levels during normal university weekday activities (e.g., lectures and labs), but they did not include this feedback in their analysis because the dataset was too small to support extensive modeling of the HRV-stress relationship. Ultimately, the authors arbitrarily chose the 10th percentile as an indicator of higher-stress time intervals.

Chan et al. [61] employed a conditional probabilistic auto-regressive (CPAR) model to synthesize PPG signals starting from the Wearable Exam Stress Dataset. The authors compared the real data with the synthesized ones through statistical similarity. The value of statistic similarity mean ranges from 98.66% to 99.43%, static similarity median from 99.38% to 99.90%, and static similarity standard deviation from 92.48% to 93.74%.

### 3.3. Bias Assessment

The results of the bias assessment for each study are reported in Table 2. Of the 32 included studies, 6 [23,43,51,55,57,58] were found to be at serious risk of bias. In [23], the serious risk was due to the low rate of labeled data: 471 out of 1,737,625 total heart rate data points. Despite adopting a semi-supervised learning technique to predict the stress label, the authors should consider the large quantity of unlabeled data when assessing the risk of bias. In [43,51,55,57,58], the serious risk of bias was related to the interventions’ classification domain because, as discussed in Section 4, the authors exposed the participants to an activity with an arbitrarily labeled stress level. For instance, the authors of the study reported in [57] arbitrarily labeled the stress level of students, assuming that a low or high level of stress would result in a performance below 70% on exams. The authors labeled the stress level of students who achieved a score greater than or equal to 70% of the total score as “moderate” and the stress level of the other students as “low or high.”

In [58], the serious risk of bias could be attributed to outcome measurements. The authors did not clarify how the outcomes were labeled and, consequently, how the metrics were computed.

Fourteen of the included studies [23,25,32,34,36,42,43,44,45,61,62,63,64,65] were assessed as being at moderate risk of bias in at least two of the four domains. The domains most exposed to risk of bias are the classification of interventions and the measurement of outcomes. The study reported in [29] was found to lack information in the domains of intervention classification and missing data. This study presented an English web-based learning platform, and the participants were not exposed to any interventions.

### 3.4. AI Algorithms

**Figure 4:**
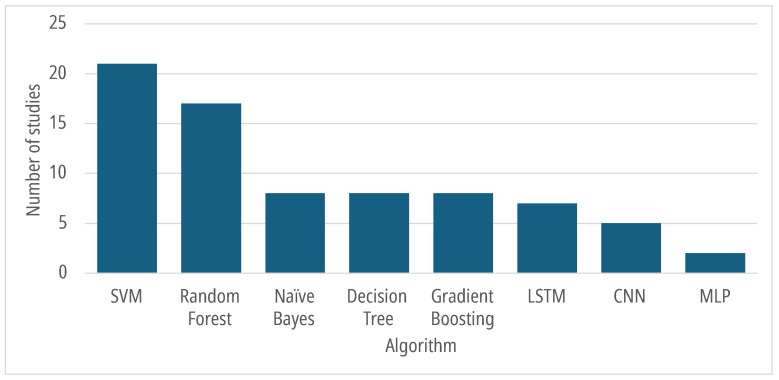
Number of included studies by applied algorithm.

Figure 4 shows the number of studies for each of the main AI algorithms.

Most of the considered studies applied the most common machine learning and deep learning algorithms. The SVM algorithm was the most frequently used, primarily for stress detection [23,43,47,51,54,57] and attention level detection [25,30,36,37,53]. The accuracy metric related to the SVM algorithm in the considered studies ranged from 60% in the stress level analysis proposed in [57] to 100% for critical thinking detection [49] and 98.08% for the attention level detection in [30]. The random forest algorithm was primarily used for stress detection tasks [40,43,50,51,54,59] and attention level detection [25,36,53]. Aguilar-Herrera et al. [40] did not use the random forest for the classification task, but adopted a random forest regression for feature selection instead. The accuracy metric related to random forest was minimal in Prieto et al. [32], who attempted to classify the social plan of interaction during a lesson, i.e., to discriminate between the moments in which the teacher is interacting individually, in a small group, or with the whole classroom of students. In this task, the random forest algorithm achieved an accuracy rate of 67.3%. The best accuracy metric for random forest is 99.98% for stress classification in the work of Chandra et al. [50].

In [49], the authors detected critical thinking through a task consisting of recognizing false information, with an accuracy of 100% using SVM, naïve Bayes, and decision trees. The accuracy of naïve Bayes ranges from 74.5%, as reported in the stress detection study, conducted in [54], to 100%, as reported in [49]. The accuracy range of decision trees varies from 65% in predicting success in answering a question [48] to 100% [49]. Some of the reviewed studies applied several types of gradient boosting, including light gradient boosting machine (LGBM) [52] and eXtreme Gradient Boosting (XGBoost) [37,42]. The accuracy of gradient boosting methods ranges from 90.6% (gradient boosted tree) for predicting the social plan of interaction, as discussed in [32], to 99.9% (boosted regression tree) for the concentration detection task reported in [39].

In [34], the authors adopted an original approach using a hybrid deep learning algorithm that integrates LSTM and CNN, as described in [67]. In this hybrid algorithm, the LSTM was used to process physiological data, then accelerometer and gyroscope data were processed by a CNN algorithm. The final step combined the processed data by applying a fully-connected layer. In [24], the authors used StressNeXt [68], an algorithm for stress and emotion recognition consisting of a convolutional block followed by four multi-kernel blocks, to analyze data from chest-worn devices.

In their work, Patil et al. [58] used a distributed machine learning (DML) approach, whereby the machine learning algorithms were computed across different computing nodes. Furthermore, the authors employed a residual (ResNet) network algorithm for the classification task. This network contains residual blocks that allow the input to be directly connected to the output of the block.

Wu et al. [60] developed an Emotion Recognition Multi-Attention Model (EmoMA-Net) that includes an input block comprised of biosignals, a convolutional neural network (CNN) module for feature extraction, a memory-attention block comprised of a time series memory system (TSMS) module, and a prediction block comprised of a custom voting classifier.

### 3.5. Devices and Collected Data

**Figure 5:**
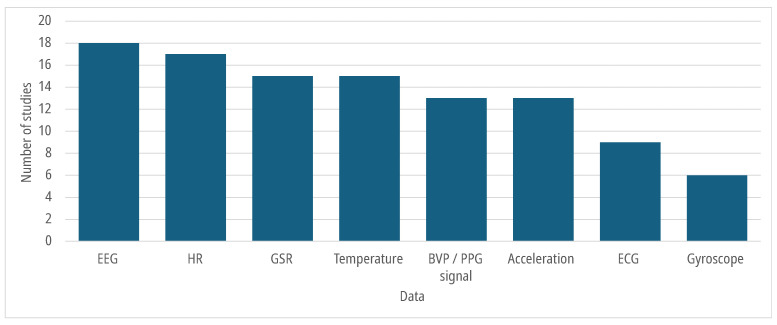
Number of included articles by collected data.

Figure 5 shows the number of studies for each signal. The included studies were divided into 11 different groups, depending on the signals collected, as shown in Table 3.

**Figure 6:**
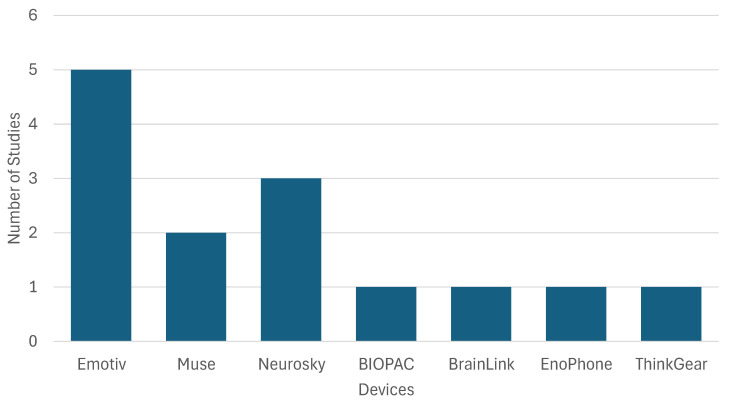
EEG devices used across studies.

In the selected studies, EEG was used to measure neurophysiological activity related to learning, attention, working memory, and mental fatigue [24,26,28,29,31,32,33,36,37,38,40,48,49,50,53]. Data collection involved different wearable devices (Figure 6), with the Emotiv EPOC and EPOC+ used in [26,28,33,48,53]. These headsets include 14 electrodes placed according to the international 10–20 system and sample brain activity at either 128 Hz or 256 Hz. Muse, a low-density EEG system with four frontal and mastoid electrodes, was used in [31,49] and offers higher comfort at the cost of lower spatial resolution. The NeuroSky MindWave device, used in [24,32,37], provides only one frontal channel and is limited to basic brainwave monitoring. In [33], the BIOPAC system was used, featuring five electrodes (Fz, F3, F4, C3, C4), which allows better coverage of prefrontal and motor-related areas. Other systems such as BrainLink [38], Enophone [40], and ThinkGear [36] were also used, often relying on proprietary SDKs that generate attention or relaxation indices rather than raw EEG traces.

Since Emotiv, EPOC or EPOC+ feature more than 10 channels coverage at 128–256 Hz, allow access to raw signals, and are compatible with EEGLAB or MATLAB, these devices were likely used when authors targeted region-specific indicators of focused attention and working memory [28,53], modeling of self-regulated learning with explainable features [26], comparison of 2-D and VR conditions where frontal activity mattered [33], inference of perceived difficulty and correctness in an ITS [48], and stress classification using time–frequency features across sites [50]. The Muse device affords a quick setup, increased comfort, and stable frontal recordings, which are useful properties in classroom-adjacent tasks [31,49]. The BrainLink devices allow for low-burden recordings, with built-in attention or relaxation indices, and were paired with PPG to capture immersion differences between VR and online video while keeping hardware simple [38]. Several studies paired or substituted EEG with Empatica E4 to track EDA and BVP as proxies of sympathetic activation with good motion tolerance and easy wear, either to complement EEG in stress or affect models [26,50] or to operate during classes and contests when headsets were impractical [41,51,52], while multi-channel systems offer spatial granularity for cognitive assessment, low-density or wrist devices improve comfort in learning settings.

Studies in the second group used the Empatica E4 wristband to analyze participants’ GSR signals during the task. Additionally, the experimental setting of the study reported in [26] included an eye tracker (Tobii Pro Nano) and timestamped navigational logs to measure affective and motivational states of the participants during essay reading and writing. In [36], the authors recorded participants’ activities through an Intel RealSense D455 depth camera, and GSR signals were processed using a Gaussian low-pass filter in the Bio-SP toolbox embedded in MATLAB to predict the attention level of 18 students watching a learning video. In [50], the investigators collected EEG, GSR, Skin Temperature, and HR signals to detect participants’ stress during the MIST test. The GSR and skin temperature signals were filtered using a Savitzky-Golay filter, and the heart rate (HR) signal was upsampled to 4 Hz. The authors of [26] used the Python PyEDA toolkit to preprocess the GSR data and downsampled the BVP readings to 4 Hz.

In [32], a teacher was monitored through eye tracking, including an audio-video stream and smartphone acceleration data, for each of the four classes settled by the authors to extract classroom orchestration graphs. In [38], VR glasses (Pico Neo 2 Smart) were used to watch a video about the city of Guilin, China, and a finger-clip blood oxygen probe (KS-CM01) was used to collect PPG signals to evaluate the learning immersion experience of the involved students. The PPG signal was then filtered, and the fast Fourier transformation (FFT) was applied to obtain a signal with a prominent amplitude near 1 Hz.

The authors of [48] collected ECG and EMG data through the SHIMMER sensor during an English test. Their aim was to evaluate perceived difficulty and predict success. The four ECG electrodes were located on the lower ribs and clavicle, and the three EMG electrodes were all located on the upper trapezius muscles. Baseline wander was removed from the ECG signal using a median filter with a 200 ms window. This was followed by applying a median filter with a 600 ms window, and by subtracting the filtered signal from the original signal. The signal was then filtered through a bandpass filter between 0.7 Hz and 20 Hz. The EMG signal was preprocessed by removing peaks within 3% of the signal’s minimum and maximum values. Then, it was filtered through a third-order Butterworth finite impulse response (FIR) low-pass filter with a cutoff at 0.4 Hz. Finally, it was normalized in the range [0,1].

In [24], PPG and ECG signals were collected. During the task, which was based on solving Sudoku exercises, the authors aimed to assess the stress level of the students, who were equipped with a Polar Variety Sense to record the PPG signal and a BMD101 to record the ECG signal. A Butterworth bandpass filter was used: one between 0.5 Hz and 5 Hz for the PPG signal, and one between 5 Hz and 15 Hz for the ECG signal.

Most EEG studies applied signal preprocessing to improve signal quality. In [26,28,29,31,32,33,37,38,40,48,49,50,53] raw signals were cleaned using a bandpass filter typically set between 0.5 Hz and 40 Hz. For instance, [48] used a Butterworth filter between 0.4 Hz and 65 Hz, while [50] used a fifth-order filter between 0.5 Hz and 30 Hz. Study [24] filtered the EEG between 0.1 Hz and 15 Hz to retain slow frequencies linked to stress detection. In [36], EEG preprocessing was skipped; the authors directly used precomputed attention scores from the ThinkGear platform. ICA was performed in [26,28,33,48,50,53] to isolate blink or muscle artifacts. In [24], EEG signals were resampled to 256 Hz to match the sampling frequency of ECG and PPG sensors for multimodal alignment.

**Figure 7:**
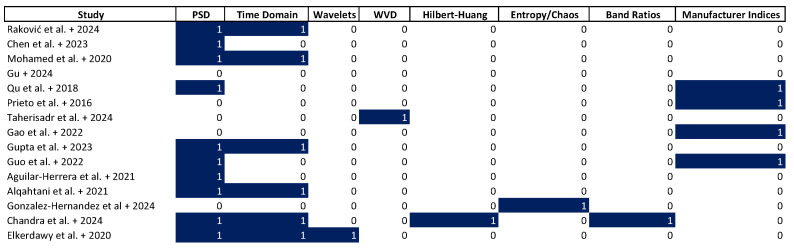
EEG signal characteristics (features) extracted per study. The studies involving EEG signals are: Raković et al. (2024) [26], Chen et al. (2023) [24], Mohamed et al. (2020) [28], Gu (2024) [29], Qu et al. (2018) [31], Prieto et al. (2016) [32], Taherisadr et al. (2024) [33], Gao et al. (2022) [36], Gupta et al. (2023) [37], Guo et al. (2022) [38], Aguilar-Herrera et al. (2021) [40], Alqahtani et al. (2021) [48], Gonzalez-Hernandez et al. (2024) [49], Chandra et al. (2024) [50], and Elkerdawy et al. (2020) [53].

Feature extraction focused on frequency-domain indicators of engagement and fatigue. Figure 7 illustrates the distribution of EEG feature extraction methods across, highlighting the specific techniques applied in each case. Power spectral density (PSD) was computed in [26,28,31,37,38,40,48,50,53], often using FFT or Welch’s method, to estimate power across δ, θ, α, β, and γ bands. Time-domain features such as mean, standard deviation, skewness, and Hjorth mobility and complexity were extracted in [28,37,48,50,53]. In [53], wavelet decomposition was used to extract energy coefficients linked to working memory. More complex decompositions were applied in [33] with smoothed Wigner–Ville distributions (WVD) and in [50] using Hilbert-Huang transforms to isolate task-related frequency shifts. In [49], chaotic measures such as Lyapunov exponents, sample entropy, and fractal dimension were used to describe cognitive engagement during problem-solving. Band ratios, such as α/β, were reported in [50] to estimate stress or attentional shifts. Studies [31,38] relied on manufacturer-supplied indices for attention and relaxation, without additional raw data analysis.

Multimodal EEG setups were described in [24,29,32,36], combining brain signals with physiological data (e.g., ECG, PPG) or behavioral recordings (e.g., screen interactions). In [29], EEG was synchronized with keystroke dynamics and response time during quiz solving; in [24], EEG was merged with ECG and PPG to classify stress scenarios during a math task. Study [32] recorded EEG and paired it with smartphone sensor data during learning in transit, while [36] used EEG-derived scores to segment user behavior in an educational video.

EEG devices differed in spatial granularity, portability, and the type of data they provided. Multi-channel systems (Emotiv, BIOPAC) allowed spatial mapping of brain regions, enabling the extraction of region-specific cognitive features. In contrast, single-channel devices (NeuroSky, BrainLink, ThinkGear) only offered frontal lobe information, limiting their use to coarse attentional metrics. Some studies prioritized comfort and real-time feedback (e.g., Muse, ThinkGear), while others opted for higher resolution and richer features (e.g., Emotiv, BIOPAC) suitable for offline analysis. Signal quality, preprocessing depth, and feature types varied substantially across studies, reflecting both technical constraints and the diversity of learning contexts investigated.

In [47], the Bitalino sensor was used to collect ECG and GSR signals during an oral presentation and a written exam to assess the stress level of 10 students of electrical engineering. In [54], the Empatica E4 was used to collect the ECG signal, and 24 HRV features were extracted using Kubios Scientific Software to identify physical, social, and cognitive stressors in a sample of 26 university students. In the study reported in [64,65] ECG data were extracted from the WESAD dataset and processed through the empirical mode decomposition (EMD) algorithm. Awais et al. [56] resampled the collected data at 200 Hz and used 4 s windows with 50% overlap. In [44], the authors aimed to detect the emotions of 8 students. The students wore a Xiaomi Mi Band 1S to capture heart rate (HR) and step count data and logged their emotions on a smartphone.

Some studies [27,34,42,46] used smartwatches to collect HR and acceleration data. Participants were also given a smartphone to report their emotional state or activities through a mobile app. The authors of [25,45] used different devices to collect HR and acceleration data. In [46], Herrera-Alcantara et al. implemented the discrete wavelet transformation (DWT) algorithm in Java using orthogonal filters, while Tanaka et al. used the sliding window method for the feature extraction in [25]. The authors of the studies that collected HR and acceleration data had different goals, ranging from concentration estimation [25], to activity recognition [27,34,46], to emotional state detection [42], and subjective difficulty detection [45].

In [41], 31 university students were equipped with Empatica E4 smartwatches during each class to evaluate their perceived satisfaction, usefulness, and performance, collecting GSR, BVP, HR signals, and skin temperature. The authors extracted histogram-based features, the first five Fourier transform coefficients, the root mean square of the signal, and the first five autocorrelation coefficients. Egilmenez et al. [43], aiming to detect stress during stressful tasks, provided participants with an LG Watch Urbane 2 smartwatch, a custom-built smartwatch based on the Northwestern-developed NUSensor platform for GSR data collection, a Polar H7 chest strap for HR data collection, and a NEULOG GSR to test the reliability of the two wrist-worn sensors. Two approaches were used for feature extraction: an event-based method considering the start and end times of each task and a minute-based method using overlapping windows. Correlation-based feature subset evaluation (CFSubset) was used for feature selection.

In [23,30,39,51,52], smartwatches were used to collect HR, acceleration, and GSR data with different aims, for either attention level detection [30,39], stress detection [23,51], and dataset creation [52]. Islam et al. [23] employed an unsupervised learning technique, based on a series of progressively fine-grained sieves. Can et al. [51] developed a preprocessing tool to remove artifacts, setting the percentage threshold between the data and the local average to 20%. The authors employed MATLAB built-in tools, along with Marcus Vollmer’s HRV toolbox, the Fast Fourier Transform (FFT), and the Lomb–Scargle periodogram, for the feature extraction.

Laporte et al. [52] extracted HRV features by analyzing the BVP signal. They followed these steps: (1) Signal segmentation into two-minute windows with a 50% overlap. (2) Third-order low-pass Butterworth filtering with a cutoff frequency of 5 Hz. (3) Moving average filtering of 16 samples. (4) Applying a peak detection algorithm. (5) Removing the peak-to-peak outside a certain range.

Hoang et al. [62] extracted physiological data from the WESAD dataset. The dataset split each subject’s data into 20 min of baseline and 10 min of stressful tasks. To avoid the imbalance, the authors used different window shifts for baseline and stress data. Regarding GSR data collected from public databases, the authors of the studies reported in [57,63] applied the cvx-EDA function to perform tonic-phasic separation.

### 3.6. Multi-Model Approach

Some of the considered articles adopted a multi-model approach, consisting of the adoption of several strategies to face detection and prediction tasks. In [44], for instance, the authors proposed three different models. In the general model, the SVM received feature vectors that the user had manually labeled. The personal model initially coincided with the general model, and then was adapted to the user by incorporating their feedback about emotion predictions. The fusion model provided a combination of the two models, obtaining the best result in terms of accuracy, with a little improvement compared with the personal model.

**Figure 8:**
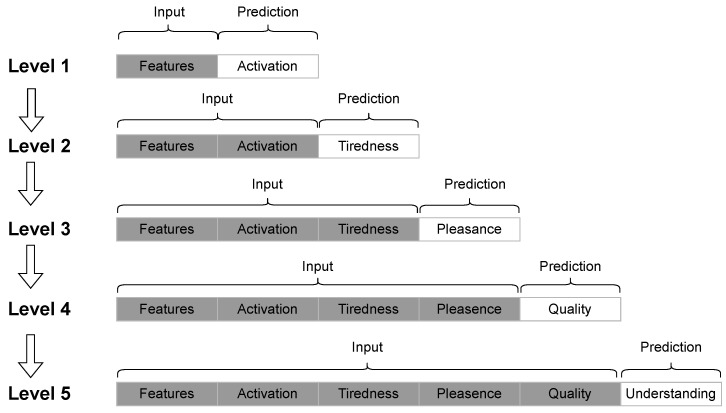
Graphical summary of the chain model described in [42], adapted from Figure 6 of the original paper.

The study described in [42] aimed to detect the level (from 0 to 2) of five different emotions (activation, tiredness, pleasant feelings, quality of the presentation, understanding of the presentation) using three different strategies. The first strategy led to five different single-output classifiers, training the classifiers for each emotional state taken independently. This strategy, called “One vs. one”, broke down multi-class problems into multiple binary classifications and was used for stressors identification, the same approach used in [54]. In the second case, a unique classification model was trained to detect at the same time levels of all five emotions. Eventually, the authors implemented a chain strategy (outlined in Figure 8), sorting the emotional states. Initially, input features were used to predict the activation level. In the next step, input features along with the predicted activation level were used to predict the tiredness level. After predicting activation and tiredness, the authors continued the chain with pleasant feelings, quality, and finally understanding.

In [48], the authors validated a linear SVM classifier with two different classification methods to detect a learner’s affective state during an English test. The first approach consisted of a *Leave-One-Out* (LOO) cross-validation, in which the algorithm was trained and tested multiple times, each time using one sample for testing and the remaining ones for training. Then, the authors applied a *Leave-One-Subject-Out* (LOSO) cross-validation, in which for each iteration one subject was used for training and the remaining ones for testing. LOSO shows the best performance in terms of F1-score. A similar approach was adopted in [54] to detect physical, social, and cognitive stressors.

To detect break moments from classes, in [52], two different prediction models were proposed: instance predictions and voting predictions. In Instance Predictions, each user was treated as independent from the group. In the Voting Prediction, predictions of all students were aggregated to formulate an overall prediction; in this case, a threshold was set. For instance, if the prediction suggested that the section is a “break” for at least 20% of students, the whole section was classified as a “break”.

### 3.7. Datasets

Some of the included studies used public datasets for training AI models. For instance, in [60], the authors presented a multimodal neural network model for emotion recognition, validating it on the WESAD dataset; a similar process took place in [68] for StressNeXt validation. WESAD, introduced in [69], was designed specifically for stress and affect detection and provides many features from 15 graduate students. Features collected in WESAD were: Accelerometer features, ECG and BVP, GSR, EMG (electromyography), respiration features, and skin Temperature. WESAD includes four emotional states: baseline, stress, amusement, and meditation. The sample consisted of 15 subjects, aged between 24 and 29. Hoang et al. [62] used two out of these states (baseline and stress) to train a baseline XGBoost model on 12 subjects and fine-tuned it on the remaining three subjects. In contrast, in [64,65], all of the emotional states reported in WESAD were classified.

In [55,57], the Physionet dataset, introduced by Amin et al. [70], was used to predict students’ grades based on their stress levels. Meanwhile, Chan et al. [61] used the Physionet dataset to perform data augmentation on the PPG signal. In [56], the CASE dataset [71] was used to train an LSTM emotion recognition model, to be included in an Internet of Things (IoT) framework for healthcare and distance learning in COVID-19. The dataset includes four emotions: amused, bored, relaxed, and scared. These emotions were collected from 30 participants during a video session.

In [59], the authors presented a multitask learning approach for predicting mental health states. Rather than training each model three times, they trained each model once to predict the three investigated states (depression, anxiety, and stress) simultaneously. The authors also performed a time-lagged version of the task, considering the variation of mental states over time. To accomplish this, the authors used the NetHealth dataset, which was collected in [72] and contains data from participants’ responses to a psychological scale called the Perceived Stress Scale (PSS) [73].

Alchieri et al. [63] used three datasets to test emotion recognition models from GSR data across different contexts. The considered databases were: The SEED dataset, introduced in [74], which contains data from 24 students, nine teachers, and 41 lectures from university context; the APSYNC dataset [75], which includes data from ten conference attendees and nineteen conference presenters; and the Workplace dataset, introduced in [76] to perform automatic recognition of breaks and work activities from data collected from fourteen academic workers. In [37], the authors used a dataset collected in [77] and retrieved from the KAGGLE repository. The KAGGLE dataset contains features of the EEG signal of 10 students, watching ten videos each, and was used to train models for student attention detection. Then, models were trained with a dataset collected by the authors, who asked 100 participants to watch different video sessions, for a total of 500 videos.

In [52], the LAUREATE dataset was presented. LAUREATE is fit for training a model for personalization in an educational context, as it contains a large number of features. This dataset reports data collected over 13 weeks in an academic setting. The authors collected GSR, BVP, Acceleration features, Skin Temperature, students’ performance, self-reported lifestyle, and study habits. The authors used the data for multiple objectives: User/device recognition, Class vs. break recognition, and self-reported affect and mood state estimation. In [27], the authors created the WLA4ND dataset, collecting data from young adults with neurodiversity and reporting six labels: Read, Write Q&A; Write; Type; Off-Task; Rest. This dataset was created for Human Activity Recognition to provide inclusive educational programs for people with neurodiversity.

### 3.8. Personalized Learning

This section investigates the potential applications of wearables combined with AI for personalized learning (RQ2). Chiefly, in response to RQ1, as shown in Table 1, the majority of the examined studies proposed algorithms that achieved favorable metrics, thereby demonstrating their effectiveness in achieving objectives. Some of these articles mention possible developments in terms of personalization.

For instance, in [28], the authors stated that their system to detect cognitive skills can provide useful information to teachers and instructors to personalize the learning process. In [37], the authors implemented a model to identify students’ attention, considering as input EEG data. First, the authors applied feature selection algorithms, finding that α and β are the most relevant frequency bands. Then, machine learning algorithms were used along with clustering methods (K-means and hierarchical), the authors stated that combining these different approaches can help in the development of a personalized e-learning system.

In [48], the authors introduced a setup in which the biometric data of students were assessed by ML models to detect their perceived difficulty level during an English language test. The authors suggested that the described system could be useful in the development of an ITS (intelligent tutoring system), a system designed to provide immediate feedback to learners, personalizing their experience.

Patil et al. in their work [58] presented a system for the real-time analysis of student behavior during exams based on gaze patterns, typing speed, hesitation intervals, heart rate, and GSR. The authors claimed that their system could improve personalized learning interventions. However, they did not detail the target value or the labeling process. This leads to a high risk of bias, which could affect any personalization-oriented applications. The authors of the studies reported in [59,60] mention didactic personalization frameworks as a potential application of their work on stress prediction and emotion recognition.

### 3.9. ERUDITE

The study report in [33] presented a personalization approach based on reinforcement learning. ERUDITE aimed to adapt the learning experience to the learner’s state, through a reinforcement learning algorithm called Q-learning.

The system collected EEG data of the learner to provide binary classifications of their learning state (LS), drowsiness state (DS), and sickness state, based on the simulator sickness questionnaire (SSQ), for a total of eight ($2^{3}$) possible combinations, as shown in Table 4.

A total order (≺) was defined on student states, where $s_{8}$ was considered the best state and $s_{1}$ the worst one, so that $s_{1}≺s_{2}≺\ldots ≺s_{7}≺s_{8}$. The ERUDITE system provided students with a didactic presentation that could be displayed in 3D using virtual reality (VR). The system offered five possible actions to enhance the learning experience.

- $a_{1}$: Give a break.
- $a_{2}$: Enable VR (Virtual Reality).
- $a_{3}$: Disable VR (Virtual Reality).
- $a_{4}$: Changing the content of the presentation.
- $a_{5}$: No change.

Equation (1) describes the Reward r(s,a), related to state *s* and action *a*:

(1) $$r\left(s,a\right)={\mathrm{Quiz}}_{\mathrm{grade}}+{\mathrm{State}\mathrm{Improvement}}_{s\to s^{\prime }},$$

where $\mathrm{State}\;{\mathrm{Improvement}}_{s\to s^{\prime }}>0$ if $s≺s^{\prime }$ and <0 otherwise. The goodness of action *a*, taken by the model, was determined by the value of Q(s,a), which was periodically updated as in Equation (2):

(2) $$Q\left(s,a\right)\leftarrow Q\left(s,a\right)+\alpha [r\left(s,a\right)+\gamma \max_{a^{\prime }}Q(s^{\prime },a^{\prime })-Q\left(s,a\right)],$$

where $s^{\prime }$ is the state occurred after action *a*, α is the learning step size and γ is called discount factor. The ϵ-greedy policy, which could be applied to this method, consists of defining a small probability ϵ to explore different scenarios, choosing a random action.

The authors measured an increase in the spectral power of the high-frequency sub-bands (10–25 Hz) of the EEG signal of the students when they transitioned from a traditional 2D learning environment to an immersive VR framework. Performance in solving the questionnaires administered at the end of the lessons increased alongside the spectral power of the high-frequency sub-bands.

### 3.10. Online Processing

ERUDITE system is based on an IoT human-in-the-loop framework: the input data (state of the student) is analyzed by an RL algorithm, alongside the reward provided by the human performance in a quiz and by the comparison between the previous state and the current one, as shown by Equation (1). Then, the student receives a new suggested action, giving another feedback. Awais et al. [56] proposed an ultra-reliable and low-latency IoT framework for student learning and healthcare in situations such as COVID-19. Authors improved the low-latency deterministic protocol through the use of shared slots (SSs), reducing the probability of failure.

Gu [29] proposed an IoT framework based on a fog-based cloud storage to enhance the monitoring of the student’s health information by the teacher. Other works presented real-time data exchange. For instance, in [44], wearable devices, smartphones, a cloud system, and internal servers were connected. The student’s biometric data (heart rate and step count) was downloaded from Google Cloud, preprocessed on internal servers, and then used by the AI model to detect the student’s emotions. The outcome of the model was displayed on a mobile app on the student’s smartphone. The student could then tag the outcome if it did not match their emotional state. This feedback loop allowed continuous retraining and improvement of the model. In a similar way, in [34], students were notified on their mobile phone when their heart rate patterns were considered unusual for school hours. Students who received the warning had to specify their current activity.

## 4. Discussion

The main purpose of this review was to investigate how AI techniques are applied to biometric data recorded by wearable devices in educational contexts. The outcome of this analysis suggests that this is a rapidly expanding field, where technological innovation aids educational needs in enhancing personalization, engagement monitoring, and stress management. Previous reviews examined the contribution of wearables in the educational setting without focusing on the role of AI algorithms [8,78,79]. The role of AI algorithms in personalized learning was also investigated in contexts where no wearable devices were involved [80].

The combined use of wearables and AI algorithms, which are mainly used to detect stress and attention levels, can also help teachers understand each student’s needs and provide personalized support. Ahmed and colleagues [81] have shown how LLM and AI can help provide tailored recommendations based on students’ biometric data, reducing teachers’ administrative pressure, and workload in trying to give personalized feedback. AI algorithms could provide teachers with feedback based on the biometric monitoring of their students. For instance, if high stress or low attention levels are detected, teachers could be advised to provide additional guided exercises to simplify the material or provide lecture notes prior to the lecture. This can help teachers tailor their teaching to individual students, which is an important skill in today’s classroom.

Compared to earlier reviews, the present work provides a broader and comprehensive analysis of applications, technological capabilities, methodological approaches, and educational implications.

### 4.1. Strengths, Weaknesses, and Future Opportunities of the Considered Studies

The results of the numerous contributions that were examined prove that the combination of wearables and AI algorithms is effective in education, primarily for classifying mental states like stress or attention. Wearable devices were used for different purposes. Stress detection remains one of the most frequent applications [24,47,50,51,62], due to the widespread concern of academic stress in post-pandemic contexts [7]. The detection of the subject’s level of attention and cognitive monitoring is also frequent, with EEG and physiological data used to measure engagement, concentration, or cognitive skills [9,28,30,37,53,63]. Other works introduced wearable devices for classroom orchestration [10], emotion recognition [42,44,60], and personalized learning systems [33].

Despite this variety, the applications remain centered on individual monitoring tasks, with relatively limited exploration of collaborative or social learning contexts. Few studies examined neurodiverse learners [23,27] or the potential of wearables to foster inclusivity in education. Moreover, although the field of intelligent tutoring systems is beginning to intersect with wearable technologies [33,48], systematic integration into adaptive pedagogical frameworks is still at an early stage. By broadening the scope of wearable applications beyond stress and attention, increasingly sophisticated and collaborative educational processes could be developed.

Among the contributions examined in this review, there is a considerable diversity of wearable devices, ranging from commercial wristbands such as Fitbit and Empatica E4 [23,25,30,39,52] to more specialized EEG headsets like Emotiv EPOC, Muse, and NeuroSky [26,28,31,37,49]. These devices allow the simultaneous collection of multimodal signals, including EEG, ECG, GSR, BVP, and inertial data from accelerometers and gyroscopes [24,36,38,50]. The integration of multiple data sources provides richer insights into students’ cognitive and emotional states, as demonstrated in studies that combined EEG with ECG and GSR to detect stress and attention [24,48,50]. The majority of studies in which the authors collected the data themselves (25 out of 32) employed fewer than three devices, demonstrating the reliability of less complex setups. As for the databases used by the authors of the other 11 articles included in the review, 7 out of 8 databases used fewer than two devices.

Some technical aspects should also be noted. First, real-time data processing is seldom explored, with most studies relying on offline analysis. Additionally, only a few contributions proposed hybrid deep learning approaches (e.g., LSTM+CNN) for multimodal integration [34,58] or adaptive reinforcement learning systems for personalization [33]. Lastly, while wearable devices acquire large quantities of continuous data, little work has been achieved in integrating these data within broader IoT or edge-computing infrastructures [33,56], which would be instrumental to scale up classroom-level deployment. These gaps suggest opportunities for future work to leverage more advanced architectures and computational models. Among the studies considered in this review, there is a predominance of short-term experimental setups, often designed to test stress detection [24,43,47,50,51] or attention measurement [30,36,37,39], while these contributions report promising classification results, the reliance on small samples and highly controlled experimental setups limits the degree of usability. The included studies did not report any gender/age differences in biometric data. In fact, the lack of analysis of gender/age-wise effects of the biometric data is another limitation deriving from the small sample sizes considered by the authors. Small sample sizes are also a weakness in the analysis of learning outcomes and complicate the assessment of the impact of the proposed models on students’ lives. Linking AI-wearable output to concrete educational outcomes is one of the most significant research gaps.

The mental state of the subjects was labeled with different methods. In some studies, students self-reported their states [23,41,42,44,45,46,48,52]. Other studies used tasks that are arbitrarily labeled by the authors [43,47,51]. Both approaches can be subject to bias from participants or investigators. Since the labeling is a core process in the classification tasks, an approach based on a validated test, such as MIST, Cognifit, or the Auditory Oddball test, could be preferable when a stable metric is desired. The use of self-reported data for labeling [25,30,39] further reinforces this methodological constraint, as this practice may not fully capture the dynamic and diverse experiences of students in real learning environments. Additionally, self-reports are, compared to validated tests, a less objective labeling approach, susceptible to eventually introducing unwarranted variation in the target variable. Validated tests allow a greater degree of replicability of experiments and their comparison.

Only a few studies explored the use of AI applied to biometric data for personalization purposes. A single session monitoring experiment can be used as the base for personalization, as in this way the student profile can be characterized, and the learning strategy can be planned accordingly. In addition to reinforcement learning as proposed in [33], LLMs could play a role in the future direction of research by providing students with suggestions tailored to their careers. Meng and Guo, for instance, developed an LLM-based decision system that integrated a course knowledge graph and student profile data to help the students with academic recommendations [82]. Further integration of intelligent tutoring system (ITS) technology with continuous biometric data monitoring [48] could also be explored, as it could lead to a novel approach to personalized learning. Furthermore, continuous monitoring of biometric data could also play a key role in inclusive education, as demonstrated in [23,27] for learners with neurodiversity.

Issues such as privacy, trust, and user acceptance were only marginally addressed, despite their centrality to sustainable integration of wearables in schools and universities [8,52]. The lack of attention to ethical dimensions should be of particular concern, as biometric data are highly sensitive and can significantly affect user trust. Moreover, given the recent normative developments such as the AIAct [83], future research should pay attention to strictly following regulations when using AI in education. Future research could investigate longitudinal adoption patterns, including resistance, habituation, or abandonment of wearable devices, and embed privacy-by-design principles into system development.

From the methodological standpoint, most studies relied on cross-sectional, lab-based experiments with limited sample sizes [24,37,47]. Classification tasks dominated the analytical approaches, with accuracy and F1 scores serving as the primary performance indicators, while these metrics are important, they provide limited insight into the educational impact of wearable interventions, such as improvements in learning outcomes, engagement, or well-being. Some innovative methods are emerging, including hybrid deep learning [34], multi-model fusion [42,44], and reinforcement learning for personalization [33]. Few works adopted mixed-method approaches that integrated quantitative biometric data with qualitative insights from students or teachers. Similarly, longitudinal designs were scarce, with a lack of knowledge on whether observed effects are sustained over time. These methodological imbalances limit the generalizability and practical impact of the findings.

To summarize, the included studies showed a good reliability in classification tasks, with converging results across devices and multimodal signal processing. Nevertheless, many aspects, such as reinforcement learning strategies for personalization, integration with the ITS, and ethical and legal framework development, were rarely faced and could be considered as future directions to be taken by the research.

### 4.2. Strengths and Weaknesses of This Review

This review contributes to the literature by providing the first comprehensive mapping of AI applications of wearable biometric data in education. It integrates available evidence into a multilevel framework that spans technological (devices, algorithms), individual (cognition, stress, emotions), organizational (classroom orchestration, teacher support), and societal (ethics, privacy, policy) perspectives. In doing so, it aims to advance the discussion from technical performances towards the broader issues of adoption, legitimacy, and educational value.

This review also faces limitations. The search was limited to the Scopus database, potentially omitting relevant works indexed elsewhere. Only English-language studies were considered, which may exclude important findings from non-English contexts. Moreover, the literature featured its own biases, particularly the predominance of stress and attention studies, which narrowed the understanding of wearables’ full potential in education. These limitations are acknowledged while also underscoring that they do not undermine the robustness of the review’s contributions.

Overall, the review highlights both the promises and the limitations of AI-enabled wearables in education, while significant progress has been made in detecting stress, attention, and cognitive states, ample integration in educational contexts remains limited. These findings underscore the need for interdisciplinary approaches that combine technical innovation with solid theoretical grounding, methodological rigor, and ethical responsibility.

### 4.3. Answers to Research Questions

Based on the findings of this work, we can approach the research questions asked in Section 2.

RQ1 *How can biometric data collected via wearable devices and analyzed through AI algorithms provide reliable information in educational contexts?*
Answer: The included studies show that AI applied to wearable biosignals yields reliable indicators of stress, attention, cognitive engagement, perceived difficulty, fatigue, and learning-relevant activities under classroom-proximate tasks. For stress, models trained on ECG and HRV, GSR, and PPG reached high accuracy in validated protocols and authentic settings. For attention and engagement, wearable and EEG-based models achieved strong performance during lectures, videos, and cognitive tests, and high rates for multiple instantaneous and sustained attention constructs when using EEG with traditional and deep models. Human activity recognition relevant to classroom orchestration and inclusive support also performed well. Together, the results of this review indicate that wearable biometrics analyzed with standard machine learning and deep learning can provide valid task-level information about learners’ affective and cognitive states in educational contexts, if sensing, labeling, and validation are implemented carefully.
RQ2 *How can these frameworks enable continuous personalization in education?*
Answer: The review identifies several information types that are both detectable with wearables and directly actionable for continuous personalization. First, stress load and arousal derived from ECG or HRV, GSR, and PPG can guide pacing, breaks, and task sequencing during activities (e.g., moving from high-pressure tasks to relaxation when stress exceeds a threshold). Second, attention and engagement metrics inferred from EEG and wrist signals are suitable for dynamic difficulty control and modality adjustments during lectures and videos, and for daily study monitoring that can trigger guidance in self-regulated learning. Third, perceived difficulty and success likelihood, estimated from combined EEG, ECG, and EMG during testing, can be used to time hints, adjust item difficulty, or choose feedback modality within an intelligent tutoring workflow. A reinforcement learning prototype [33] illustrates a closed loop in which EEG-based learner states trigger actions such as breaks, VR on or off, and content changes, using performance-linked rewards to converge on effective policies. Fourth, profiling and orchestration information supports both individual and group personalization: repeated EEG-based estimates of cognitive skills can inform level placement, while activity recognition and smartwatch-based analytics provide context for inclusive support in neurodiverse populations and for routine classroom management. These opportunities can enable continuous didactic personalization using signals and procedures proposed withing the studies examined in this review in educational settings.

## 5. Conclusions

In this review, we analyzed the current state of biometric data collection through wearable devices and AI algorithms applied to education. The literature highlights the effectiveness of the devices used for stress detection, emotional and cognitive state recognition, and human activity recognition.

We explored the main ML and AI algorithms used to detect stress, attention, and other cognitive measures in an educational context. Several signals were used for this purpose, such as EEG, HR, GSR, and ECG, in very different experimental setups. Some studies applied a multi-modal approach to identify the best model for detection tasks. However, we noticed a lack of standardized methodology for data collection and task design in the literature. Only a few studies explicitly pave the way for applications of wearables and AI for a personalized education.

Future research could focus on the application of wearables and AI in an educational context to obtain a personalized student’s psychophysiological profile and suggest a personalized educational path accordingly.

## Figures and Tables

**Table 2:** Risk of bias assessment for the included studies.

Study	Interventions Classification	Missing Data	Outcome Measurement	Results Selection
[25]	Moderate	Low	Low	Moderate
[26]	Low	Low	Low	Low
[24]	Moderate	Low	Low	Low
[27]	Low	Low	Moderate	Low
[28]	Low	Low	Low	Low
[29]	No information	No information	Moderate	Low
[30]	Moderate	Low	Low	Low
[31]	Low	Moderate	Low	Low
[32]	Low	Low	Moderate	Moderate
[33]	Low	Low	Moderate	Low
[34]	Moderate	Moderate	Moderate	Low
[35]	Moderate	Low	Low	Low
[36]	Moderate	Moderate	Low	Low
[37]	Low	Low	Low	Low
[38]	Moderate	Low	Low	Low
[39]	Moderate	Moderate	Low	Low
[40]	Low	Low	Low	Low
[23]	Moderate	Serious	Moderate	Low
[41]	Low	Low	Low	Low
[42]	Moderate	Moderate	Moderate	Low
[43]	Serious	Moderate	Moderate	Low
[44]	Moderate	Low	Moderate	Low
[45]	Moderate	Low	Moderate	No information
[46]	Moderate	Low	Low	Low
[47]	Low	Low	Low	Low
[48]	Low	Low	Low	Moderate
[49]	Moderate	Low	Low	Low
[50]	Low	Low	Low	Low
[51]	Serious	Low	Low	Low
[52]	Moderate	Low	Low	Low
[53]	Low	Low	Low	Moderate
[54]	Low	Low	Moderate	Low
[55]	Serious	Low	Low	Low
[56]	Moderate	Low	Low	Low
[57]	Serious	Low	Moderate	Low
[58]	Serious	Low	Serious	Moderate
[59]	Low	Low	Low	Low
[60]	Moderate	Low	Low	Low
[61]	No information	Low	Moderate	Moderate
[62]	Moderate	Low	Low	Moderate
[63]	Moderate	Low	Low	Moderate
[64,65]	Moderate	Moderate	Low	Low

**Table 3:** Description of study groups, depending on collected signals.

Group Description	Studies Included in the Group
EEG	[28,31,33,37,49,53]
EEG + GSR	[26,36,40,50]
EEG + ACC	[32]
EEG + BVP	[38]
EEG + ECG	[24,29,48]
ECG	[47,54,64,65]
ECG + GSR + BVP	[56]
HR + Step count	[35,44,59]
HR + ACC	[25,27,34,42,45,46]
HR + GSR	[41,43,55,58]
HR + ACC + GSR	[23,39]
ACC + BVP	[30,51,52]
ACC + BVP+ GSR	[62]
GSR	[57,60,63]
PPG	[61]

**Table 4:** Learner states classifications as provided in [33].

LS	1	1	1	1	0	0	0	0
**DS**	1	1	0	0	1	1	0	0
**SSQ**	1	0	1	0	1	0	1	0
**State**	$s_{8}$	$s_{7}$	$s_{6}$	$s_{5}$	$s_{4}$	$s_{3}$	$s_{2}$	$s_{1}$

## Data Availability

No new data were created or analyzed in this study.

## Abbreviations

The following abbreviations are used in this manuscript:

SVM	Support vector machine
LRCN	Long-term recurrent convolutional networks
CNN	Convolutional neural network
CRNN	Convolutional recurrent neural network
MLP	Multilayer perceptron
LSTM	Long short-term memory
DSTCLN	Deep spatiotemporal Convolutional bidirectional LSTM Network
NN	Neural network
SVC	Support vector clustering
kNN	K-nearest neighbors
NB	Naïve Bayes
SBBO	Satin bowerbird optimization
CART	Classification and regression tree
XGBOOST	Extreme gradient boosting
RBF	Radial basis function kernel
SVR	Support vector regressor
ResNet	Residual network
DML	Distributed Machine Learning
JRIP	Repeated incremental pruning to produce error reduction
PCA	Principal component analysis
LDA	Linear discriminant analysis
LGBM	Light gradient boosting machine
STResNet	Spectro-temporal residual network
TSMS	Time Series Memory System
REF–RF	Recursive feature elimination in random forest
CBAM	Convolutional Block Attention Module
CPAR	Conditional probabilistic auto-regressive model
MLR	Multiple linear regression
MTL	Multitask learning
MFCC	Mel frequency cepstral coefficients
ECG	Electrocardiography
HR	Heart rate
HRV	Heart rate variability
GSR	Galvanic skin response
PPG	Photoplethysmogram
BVP	Blood volume pulse
SpO_2_	Peripheral oxygen saturation
EEG	Electroencephalography
ANS	Autonomic nervous system
HAR	Human activity recognition
BP	Blood pressure
ICG	Impedance cardiogram
AI	Artificial intelligence
MIST	Montreal imaging stress test
TSS	Trier social stress test
WCST	Wisconsin Card Sorting Test
PSD	Power spectral density
FFT	Fast Fourier transformation
WVD	Wigner–Ville distributions
LOO	Leave-one-out
LOSO	Leave-one-subject-out
LLM	Large language model
MSE	Mean squared error
MFCC	Mel frequency cepstrum coefficient
TP	True positive
TN	True negative
FP	False positive
FN	False negative
ITS	Intelligent tutoring system
LS	Learning state
DS	Drowsiness state
SSQ	Simulator sickness questionnaire
VR	Virtual reality
IoT	Internet of Things
EMG	Electromyography

## Appendix A. Glossary

- Anxiety: The anticipation of a future threat causes muscle tension and alertness, preparing the body for danger [84].
- Attention: The behavioral and cognitive processes involved in focusing on certain information [85].
- Concentration: The ability to maintain sustained attention on a task during a certain time [86].
- Engagement: The concept refers to students who are meaningfully engaged in learning activities through interaction with others and worthwhile tasks: it involves active cognitive processes such as problem-solving and critical thinking [87].
- Stress: The non-specific response of the body to any demand made upon it [88].
